# Animal Models in Human Adenovirus Research

**DOI:** 10.3390/biology10121253

**Published:** 2021-12-01

**Authors:** Luca D. Bertzbach, Wing-Hang Ip, Thomas Dobner

**Affiliations:** Leibniz Institute for Experimental Virology (HPI), Martinistr. 52, 20251 Hamburg, Germany; winghang.ip@leibniz-hpi.de

**Keywords:** cotton rats, experimental infection, human adenovirus (HAdV), (humanized) mice, in vivo model, pigs, susceptibility, Syrian hamsters, rabbits, tree shrews

## Abstract

**Simple Summary:**

Animal models are widely used to study various aspects of human diseases and disorders. Likewise, they are indispensable for preclinical testing of medicals and vaccines. Human adenovirus infections are usually self-limiting, and can cause mild respiratory symptoms with fever, eye infection or gastrointestinal symptoms, but occasional local outbreaks with severe disease courses have been reported. In addition, adenovirus infections pose a serious risk for children and patients with a weakened immune system. Human adenovirus research in animal models to study adenovirus-induced disease and tumor development started in the 1950s. Various animal species have been tested for their susceptibility to human adenovirus infection since then, and some have been shown to mimic key characteristics of the infection in humans, including persistent infection. Furthermore, some rodent species have been found to develop tumors upon human adenovirus infection. Our review summarizes the current knowledge on animal models in human adenovirus research, describing the pros and cons along with important findings and future perspectives.

**Abstract:**

Human adenovirus (HAdV) infections cause a wide variety of clinical symptoms, ranging from mild upper respiratory tract disease to lethal outcomes, particularly in immunocompromised individuals. To date, neither widely available vaccines nor approved antiadenoviral compounds are available to efficiently deal with HAdV infections. Thus, there is a need to thoroughly understand HAdV-induced disease, and for the development and preclinical evaluation of HAdV therapeutics and/or vaccines, and consequently for suitable standardizable in vitro systems and animal models. Current animal models to study HAdV pathogenesis, persistence, and tumorigenesis include rodents such as Syrian hamsters, mice, and cotton rats, as well as rabbits. In addition, a few recent studies on other species, such as pigs and tree shrews, reported promising data. These models mimic (aspects of) HAdV-induced pathological changes in humans and, although they are relevant, an ideal HAdV animal model has yet to be developed. This review summarizes the available animal models of HAdV infection with comprehensive descriptions of virus-induced pathogenesis in different animal species. We also elaborate on rodent HAdV animal models and how they contributed to insights into adenovirus-induced cell transformation and cancer.

## 1. Introduction

Animal models are of the utmost importance for basic and applied research to not only expand and verify in vitro findings, but also to study diseases and therapies in physiological settings. In infectious diseases research, they contributed to fundamental insights into disease pathogenesis, and are essential to study mode-of-action and efficacies of anti-infectives and prophylactic treatments [1]. An ideal animal model recapitulates a particular disease or condition in a non-human organism in terms of key phenotypic, pathophysiologic, and histopathological characteristics, as well as response to treatment [2,3]. Animal models have greatly improved our knowledge on the course of viral infections including disease, associated pathology, persistence, and viral transformation in human adenovirus (HAdV) infections [4,5]. Moreover, preclinical evaluation of antiadenoviral treatment options, adenovirus-based therapeutics, and vaccines rely on data from experiments in relevant in vivo settings, as is happening currently with COVID-19 vaccines [6]. Here, different HAdV types and simian adenoviruses serve as excellent vaccine vectors, and are used to potentially fight off zoonotic diseases such as AIDS, malaria, and Ebola, besides COVID-19 [7]. In vivo studies on adenoviral vectors in general and their use in vaccine- and gene therapy approaches in particular have been extensively reviewed. The same applies to oncolytic adenoviruses in animal models that are not part of this work, but were reviewed by others recently [8,9,10,11,12]. This review outlines the current knowledge on HAdV susceptibility of different animal models, highlighting key features, strengths, and limitations. We set the focus on studies that predominantly report adenovirus pathogenesis and adenovirus-induced cell transformation.

## 2. Main Text

### 2.1. Adenovirus Disease in Humans and the Importance of Animal Models in Adenovirus Research

HAdVs belong to the genus Mastadenovirus, in the virus family Adenoviridae. They are grouped into different species (A to G), and subsequently classified in more than 100 different types, based on viral sequences and serological data [13]. HAdV infections of humans are common, with high seroprevalences worldwide [14]. These non-enveloped, double-stranded DNA viruses have genome sizes of approximately 24–48 kb, depending on the type [15].

Clinical symptoms in infected individuals vary, and there are currently no widely available vaccines nor approved specific antiadenoviral compounds available to prevent and treat HAdV infections [16]. While immunocompetent individuals develop rather mild symptoms, infections of pediatric or immunocompromised patients can cause severe and sometimes lethal disease [17]. Furthermore, different HAdV types have different tissue tropisms, and infections of the respiratory, gastrointestinal, and urinary tracts, as well as the eyes, have been reported [17]. The tissue tropism largely correlates with clinical signs in humans that range from respiratory disease, conjunctivitis, and gastroenteritis of different severities. Moreover, some types are discussed to be risk factors for obesity, as extensively reviewed elsewhere [18,19]. To understand HAdV pathogenesis and infection-related consequences, researchers have been on a ~70 year-long quest for suitable animal models that phenocopy HAdV infection of humans. These models included various rodents including xenotransplanted and genetically engineered transgenic mice and hamsters, pigs, non-human primates, and other species. All these models facilitate HAdV replication and induction of HAdV disease signs to different extents, and are employed to recapitulate aspects of the human disease. However, an ideal model that mimics the human disease has not been established to date, partly due to the species-specific nature of adenoviruses. The search remains ongoing as key findings from carefully designed and well-controlled studies in animal models continue to be of great importance to biomedical research [20].

### 2.2. First Experimental HAdV Infections of Animals

**Figure 1:**
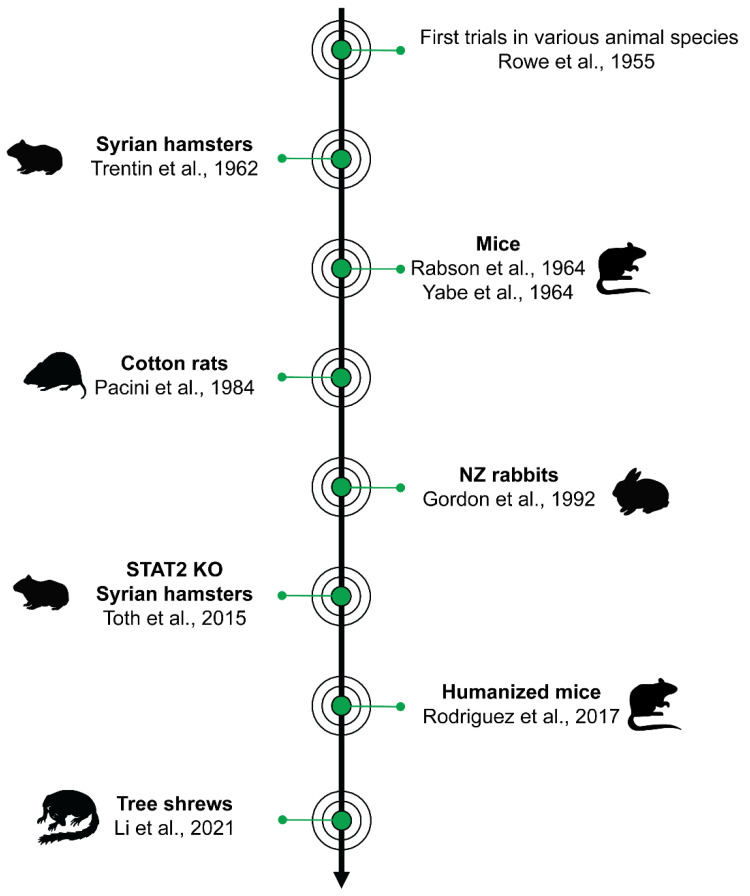
Landmark publications on animal models in HAdV research. HAdV research in small animal models was pioneered by Rowe et al. in 1955 at the NIH in Bethesda, MD (USA) [21]. That work laid the foundation for various follow-up studies and novel approaches to establish a suitable animal model to study HAdV infection, including HAdV-induced pathogenesis and cancer development in vivo. The most relevant models are Syrian hamsters [28], mice [32,33], cotton rats [37], New Zealand (NZ) rabbits [38], STAT2 knockout (KO) Syrian hamsters [39], humanized mice [40], and Chinese tree shrews [41] (sorted by publication date of the first report on the respective animal model).

The first experimental infections of animals with HAdVs date back to the 1950s and 1960s [21,22,23,24,25,26,27,28,29,30,31,32,33,34,35,36]. All of these studies confirmed findings by Rowe and colleagues, who did not observe clinical disease symptoms in experimentally infected rabbits, mice, hamsters, Guinea pigs, cats, ferrets, rats, and even non-human primates (Figure 1) [21]. Another follow-up study also reported asymptomatic persistent infection of rabbits [22]. Interestingly, they detected persistent HAdV-C5 in experimentally infected adult rabbits at 8 weeks p.i. by virus re-isolation from spleen homogenates. Subsequent approaches evaluated HAdV-induced tumors in rodents as described below [26,28,29,30,31,32,33,34,35], along with the susceptibility of dogs and even pigs to different HAdV types using different infectious doses, but none of these animals proved to be suitable to study HAdV infection [23,24,25].

### 2.3. Syrian Hamsters

(Golden) Syrian hamsters (*Mesocricetus auratus*) are medium-sized rodents that are frequently used as animal model in various fields of research. Moreover, a decent number of molecular tools and reagents are available to study host response to infection in this hamster species [42]. They are susceptible to a wide variety of DNA and RNA viruses [42], and are used as an adenovirus animal model since 1962 (Table 1) [26,27,28,29]. The Syrian hamster model is the most commonly used HAdV animal model that has successfully been applied to studies on basic HAdV pathogenesis, and especially tumorigenesis, countermeasure and (oncolytic) vector development [43]. The first records on the HAdV-susceptibility of Syrian hamsters originate from the a study performed by Trentin and colleagues, who showed that HAdV-A12 induced sarcomas over a course of 33–90 days post-infection (Figure 1) [28]. These results were confirmed by three more publications in the same year that additionally demonstrated tumor induction by HAdV-A18, as well as fatal infection of newborn hamsters with high infectious doses of HAdV-C5 [26,27,29]. Subsequent studies further corroborated these data, and also reported oncogenic properties of HAdV-B3 and B7 [30,34,44,45,46]. Much is known about the mechanisms of adenoviral tumor induction and cell transformation in vitro, but the subjects still are a matter of investigation. It is discussed that some HAdVs (such as A12, A18, B3, and B7) lead to abortive infection, and subsequently to cell transformation in hamsters, while other HAdVs replicate and cause cell pathology in these animals. Nevertheless, adenoviral cell transformation in general and HAdV-induced tumors in Syrian hamsters in particular represent an excellent system for DNA virus tumorigenesis, and built the cornerstone for various following reports on the development of adenoviral vectors that have been reviewed extensively [47,48,49,50]. Intriguingly, other hamster species have also been shown to be susceptible to HAdV-induced tumors. A single 1974 study describes HAdV-A12-induced tumor development in newborn Armenian (*Cricetulus migratorius*) and Chinese hamsters (*Cricetulus griseus*) [51].

Syrian hamsters that were used in HAdV-C5 and C6 pathogenesis studies were found to have viral titers in blood and organ samples, and serologic as well as histologic evidence of infection [52,53,54,55,56,57]. Besides HAdV-C5 and C6 replication in the lungs and other organs, the virus especially targets the liver (hepatocytes, Kupffer cells) to cause inflammation and hepatocellular necrosis, accompanied by elevated levels of liver enzymes. Weight loss as a robust clinical sign of infection has only been observed in chemically immunosuppressed Syrian hamsters that showed transiently reduced body weights upon HAdV-C5 infection [55,56]. Interestingly, male Syrian hamsters seem to be more susceptible than females, which has also been observed for other viral infections like COVID19 lately [57,58,59].

In recent studies, Radke and colleagues reported that HAdV-B14 and an emerging variant, B14p1, cause severe lung pathogenesis in intratracheally infected in Syrian hamsters characterized by local infiltrations of inflammatory cells developing into bronchopneumonia. However, although the authors did not report clinically apparent disease signs, these studies in conjunction with the studies that Tollefson and colleagues performed in Syrian hamsters allowed for comparisons of the pathogenicity of the different HAdVs and assessment of respective immune responses [55,56,60,61].

Toth and colleagues introduced the genetically modified Syrian hamster model in 2015 (Figure 1) [39]. These STAT2 knockout animals [62] show decreased interferon signaling, which facilitates higher HAdV-C5 replication, more severe liver pathology, and increased mortality when compared to wild type Syrian hamsters upon intravenous infection, and thereby resemble HAdV infection of immunosuppressed humans. Moreover, the interferon immune response was deregulated in infected STAT2 knockout hamsters [39]. Both wild type and genetically modified hamsters have been proven useful for efficacy studies on HAdV therapeutics and vaccines, as well as on vector development [43,53,54,55,63,64,65,66,67,68,69,70,71,72,73].

### 2.4. Cotton Rats

Cotton rats are small rodents that are susceptible to various human pathogens, and especially to upper respiratory tract infections where mice and rats are typically rather resistant [74,75]. The first report on hispid cotton rats (*Sigmodon hispidus*) as an HAdV animal model dates back to 1984 when Pacini and colleagues used moderate HAdV-C5 titers to infect one-month-old animals, and detected viral titers in the lungs and nasal mucosa and seroconversion as soon as six days post-infection (Figure 1, Table 1) [37]. Histopathological examinations of lung samples from infected cotton rats revealed transient peribronchial immune cell infiltration and other subtle signs of pneumonia [37]. No clinical symptoms were observed by Pacini and colleagues, but dose-dependent disease has been reported in a follow-up study, in which high HAdV-C5 doses led to more severe lung damage, and high-dose-infected animals died within the first week post-infection [76].

Cotton rats are also employed as animal models for the adenovirus-induced eye disease adenoviral epidemic keratoconjunctivitis (EKC) [78,79,80]. EKC is highly contagious, and characterized by eye inflammation and visual disturbances caused by corneal opacities; EKC outbreaks occur regularly [81,82]. Ocular HAdV-C5 and D8-infection of cotton rats show hallmarks of the human disease, including virus replication and development of the aforementioned subepithelial corneal opacities [78,79,80].

This HAdV-animal model is susceptible to HAdV infection, and even resembles human EKC. Consequently, cotton rats have been used in various approaches to test therapeutic interventions and to study oncolytic adenoviruses [65,79,80,83,84,85,86].

### 2.5. New Zealand Rabbits

Rabbits have served as models of human infectious diseases and ophthalmology for decades [87,88]. New Zealand (NZ) rabbits (*Oryctolagus cuniculus*) have been used as an animal model species for investigating persistent [22,36] and ocular HAdV infection (Table 1) [38,89,90,91,92,93,94,95]. Both papers reporting prolonged HAdV-C5 infections observed persistence of the virus in spleens for months [22,36], as well as presence of anti-HAdV-C5 antibodies in rabbit sera for up to a year post-infection [36]. The initial study on HAdV-C5-infected rabbits as a model for basic and preclinical research on EKC was published by Gordon and colleagues in 1992 (Figure 1) [38]. Through intraocular infection of female NZ rabbits, they demonstrated that several features of human adenoviral eye infection can be reproduced in this animal model, such as acute conjunctivitis, iritis, and corneal edema, followed by infiltrating immune cells at around 14 days post-infection. In addition, infected rabbits mounted a neutralizing antibody response, and confirmed previous findings that rabbits respond to infection via the humoral immune response. This ocular model of HAdV-C5 infection has been continuously used since then to assess efficacies topical use of the broad spectrum antiviral cidofovir and other treatment options against adenoviral eye infections [89,90,91,92,93,94,95]. It is very likely that NZ rabbits will serve in future studies that investigate the pathology and countermeasures against adenovirus-induced human EKC.

### 2.6. Rats

Rats (*Rattus norvegicus*) are commonly used as laboratory animal models that mimic aspects of human diseases or the human organism, especially in oncology, pharmacology, and toxicology research [96,97]. As described for other rodents above, rats also develop tumors upon HAdV infection. That was first described by Huebner and colleagues in 1963, who showed that HAdV-A12 infections induce peritoneal tumors in rats with 30% efficiency [98]. Interestingly, HAdV-A12-induced sarcomas and even retinoblastomas have been observed by other groups [32,99,100,101]. In contrast, HAdV-D9 has been shown to cause tumors in rats more efficiently, as investigated in a series of papers by Javier and colleagues. First, they showed that subcutaneous HAdV-D9 inoculation of newborn female rats led to mammary tumors during weeks to several months post-infection that exhibited three distinct phenotypes: fibroadenoma, phylloide-like tumors, and solid sarcomas. HAdV-D9-induced tumors developed, dependent on estrogen, as ovariectomized rats did not develop tumors upon infection whereas treatment with a synthetic estrogen promoted tumor formation [102]. With that, they confirmed earlier studies on HAdV-D9-related tumorigenesis [103,104]. Further analyses revealed that HAdV-D9 persisted via genomic integration in tumor cells [102]. In subsequent studies, Javier and colleagues could pinpoint the HAdV-D9 E4 ORF1 gene as the major oncogene that drives mammary tumor formation in infected rats [105,106,107]. The rat model for HAdV-infection has been used in one more study that aimed to assess the effects of HAdV-C5 major capsid protein modifications on tissue distribution within infected animals [108]. Male animals that were infected with the HAdV-C5 wild type control exhibited a hepatotropic virus dissemination with no clinical signs of an HAdV-C5-induced disease over a course of five days post-infection. Interestingly though, the study demonstrated certain penton and fiber mutations lead to viruses that are detargeted from rat livers [108].

These data indicate that rats are a good model to study HAdV-induced tumors, and possibly also adenoviral vector development, but are not suitable for pathogenesis assessment and preclinical evaluation of HAdV-vaccines and antivirals.

### 2.7. Mice

Non-transgenic immunocompetent laboratory mice (*Mus musculus*) were introduced to HAdV research in 1964, when two American laboratories concurrently showed that HAdV-A12 can induce tumors in newborn mice, comparable to those in Syrian hamsters (Figure 1) [32,33]. However, HAdV tumor induction has not been followed up upon in mice.

Comparable to the infection of Syrian hamsters, the primary target organ for HAdV replication is the mouse liver, leading to hepatocellular necrosis, and even fatal hepatitis, in HAdV-C5-infected mice, depending on the infectious dose [53,77,109,110]

Other studies reported no or only subtle viral replication, which is partly explained by the absence of the entry receptor CD46 that is used by some HAdVs [14,111,112,113]. This poses a major drawback, and prompted HAdV researchers to assess alternative models such as HAdV infection of transgenic mice [54,114,115,116] or ocular HAdV infection [117,118].

Mice are the most used animal model, with a vast variety of tools and reagents available to study nearly all aspects of host responses to infection. Thus, several studies have investigated antiviral therapies and oncolytic adenoviruses in mice [53,54,119,120,121,122,123,124,125,126], and this model will likely remain important for HAdV research.

As additional mouse models, humanized mice are routinely used to study human infectious diseases [127,128,129]. These models are generated e.g., by engraftment with functional human cells or tissues. To establish an HAdV small animal model that reproduces acute and chronic infection, Rodriguez and colleagues recently presented a humanized mouse model (Figure 1) [40]. These JAX NSG-A2 mice received HLA-A2-matching CD34+ human hematopoietic stem and progenitor cells (HSPCs), and were HAdV-C2-infected intravenously nine weeks post-transplantation. Successful infection was confirmed by the detection of adenoviral RNA in blood samples, and systemic infection was excluded for most animals, although low levels of viral mRNA could be detected in bone marrow and inguinal lymph node samples. Aside from asymptomatic infections, which are most probably due to the differing degrees of “humanization”, they observed acute infection presented as lethargy, weight loss, and even death in roughly one third of all infected NSG-A2 mice. Recorded histopathological lesions in livers of infected mice included intracytoplasmic vacuoles in hepatocytes, an increase in cell proliferation, an influx of monocytes and macrophages, and signs of fibrosis [40]. HAdV-C2 persisted in asymptomatic mice, and these mice mounted an HAdV-specific adaptive immune response.

The humanized mouse model of HAdV infection requires further evaluation, and could be a valuable alternative animal model to study HAdV persistence and reactivation.

### 2.8. Non-Human Primates

Due to a close phylogenetic relationship to humans, non-human primates are often used as models to understand infectious diseases of humans, as well as zoonotic and anthroponotic infections [130]. HAdV infection has been evaluated in non-human primate species with differing susceptibilities. First, experimental studies of various HAdV types from different species in intranasally, intracerebrally, and subcutaneously infected non-human primates were performed by Rowe et al., and failed to detect any symptoms of an HAdV-induced disease [21]. However, efficacy studies of adenovirus-based vaccines and gene delivery vectors provide evidence that non-human primates are susceptible to HAdV infection with no clinical signs of HAdV-induced respiratory disease reported to date [131,132,133,134,135,136]. Moreover, a single study described HAdV-A12-induced eye tumors in 3 out of 21 baboons (*Papio* spp.) 1 to 3 years post-infection when the virus was injected intraocularly [137].

Most recent data come from HAdV-B55 infection in tree shrews (Tupaia belangeri chinensis), squirrel-sized Asian non-human primates (Figure 1) [138]. Li and colleagues infected Chinese tree shrews intranasally, and showed that they were permissive to infection as evidenced by viral replication in the upper and lower airways and the lungs, resulting in severe pneumonia (Table 1). They additionally demonstrated that tree shrews reacted to HAdV-B55 infection with rapid seroconversion, elevated body temperatures, and upregulation of pro-inflammatory cytokines in PBMCs [41]. The tree shrew therefore poses a promising model for HAdV-induced clinical disease and research on the zoonotic potential of HAdVs that should be followed up upon, especially using other HAdVs for infection.

### 2.9. Pigs

Pigs (*Sus scrofa*) have long been excellent models for research on diseases affecting humans [139]. Many characteristics of pig anatomy and physiology resemble the situation in humans, which is why there are several advantages of the porcine model over rodents and other small animal models [140]. Their application as an animal model in HAdV research, however, has been infrequent, and can be attributed to practical reasons such as required space, costs, expense, and handling. The 1962 Betts and Jennings papers were the first to report intratracheal HAdV infection of pigs causing bronchopneumonia, accompanied by focal alveolar necrosis and lymphoid hyperplasia [24,25]. Conversely, no clinical signs were observed (Table 1) [24,25]. These initial investigations were followed up by two more recent publications, confirming the HAdV susceptibility of pigs. Intravenous HAdV-C5 injection of pigs resulted in moderate lung pathology, and viral DNA could be detected in lung, liver, kidney, and blood samples at early time points post-infection, suggesting virus replication [110]. No obvious abnormalities were detected upon histopathological examinations of liver and kidneys tissues, and clinical disease signs were absent [110]. Work by Koodie and colleagues demonstrated active replication of an HAdV-C5/B3 chimera in the lungs and spleen of intravenously infected immunocompetent pigs [141].

Collectively, these results suggest that pigs could be used as an HAdV animal model to study the disease and for countermeasure development. Future directions should include more thorough characterization of HAdV-induced pathology and immune responses in pigs.

### 2.10. Guinea Pigs

Guinea pigs (*Cavia porcellus*) are important models in human bacterial infections [142]. In addition, they are susceptible to human pathogenic viruses, such as influenza A and Zika [143,144]. An early study from 1974 describes persistent HAdV-C5 infection of guinea pigs (Table 1) [145]. After intracardial HAdV-C5 infection of male guinea pigs, Faucon and colleagues could re-isolate virus from different tissues, from blood and from spleens for long periods of times post-infection, indicating persistent infection [145]. No clinical signs or seroconversion of animals were reported in that study. A follow-up study that aimed at establishing guinea pigs as an HAdV infection model used the same virus (HAdV-C5), but at a lower infectious dose, and female guinea pigs that were infected intranasally. These animals had high viral titers and detectable viral gene expression in lung tissues early in infection, and exhibited considerable lung damage as observed by immunopathologic examination of the lungs at different time points post-infection. Moreover, the animals seroconverted to HAdV-C5 from three weeks post-infection on and persistent infections as shown by Faucon and colleagues could not be detected [146]. Notably, HAdV-induced tumor development has either not been investigated or was not reported in guinea pigs, even though they belong to the order *Rodentia*. These unresolved and even contradicting results combined with a lack of reagent availability explain why more research is needed to establish guinea pigs as an HAdV animal model. Nevertheless, guinea pigs have served in preclinical trials of HAdV-C5-based vector vaccines [147].

## 3. Conclusions and Perspectives

Basic and applied research on the various facets of HAdV infection including work in preclinical models will remain important for the next decades. Basic research will further reveal mechanisms and nuances of viral pathogenesis, persistence, and cell transformation. Applied research includes important research areas such as the testing of therapeutics and the evaluation of vector attenuation and immunogenicity, as well as (protective) efficacy of adenoviral vaccine and gene therapy vectors. Thus, the quest for an ideal small animal model that resembles key characteristics of HAdV infections will continue as, thus far, no HAdV animal model can replicate the most important aspects of HAdV infection of humans, and most available models are far from ideal (Table 2).

The Syrian hamster and cotton rat models are undoubtedly the most relevant and most utilized models to date, although they have their limitations as outlined above and summarized in Table 2. Novel approaches like the tree shrew model or infection of pigs must prove their worth.

The availability of various tools including suitable animal models to study HAdVs is particularly important as the adenoviruses are diversifying, and more and more cases of interspecies transmission pose the risk of zoonotic and anthroponotic spillover events [148,149,150,151,152,153,154,155,156]. Interesting and germane research questions that could be addressed using wild type and chimeric viruses in in vitro and in vivo studies include, but are certainly not limited to: What defines the host range of adenoviruses? How do recombination events facilitate spillover infections? How can we improve outbreak preparedness? How likely are cross-species transmission events from non-human primates to humans (or vice versa)? Notably, the non-human primate isolates belong to the genus *Mastadenovirus* in the same way as all HAdVs, and the adenovirus-induced disease in non-human primates is comparable to that in humans [157]. Similar to in humans, disease signs include mild to moderate respiratory and/or enteric symptoms, and even eye infections have been reported [157]. The field is beginning to understand aspects of host-range determinants, and both host and particularly viral factors seem to play important roles in this process [156,158]. The adenoviral DNA binding protein (DBP) is such a host range determinant, and future work will further unravel its role in HAdV cross-species transmission and adaptation of the virus to new hosts [158,159]. For all that, well-coordinated interdisciplinary research and a toolbox that includes appropriate HAdV animal models are crucial to thoroughly understand the HAdV-induced disease and its zoonotic potential.

## Figures and Tables

**Table 1:** Wild type HAdV susceptibility and wild type HAdV-induced disease in different relevant animal models. The corresponding references can be found in the respective paragraph text.

Species	HAdV Type(s) ^+^	HAdV Dose (Range) ^#^	Infection Route	Clinical Signs Of A Systemic Disease	Histopathological Lesions	Induction Of Neutralizing Antibodies?	HAdV-Induced Tumors?
Cotton rat (*Sigmodon* *hispidus*)	C5, D8, D37 and E4	10^2^–10^10^	i.m., i.n., i.o.	No *	Lung, airway and eye damage	Yes	No
Guinea pig (*Cavia* *porcellus*)	C5	10^7^–8 × 10^8^	i.c., i.n.	Not reported	Lung and airway damage	Yes	No
Humanized mouse (*Mus musculus*)	C2	1.4 × 10^4^–1.4 × 10^8^	i.v.	Lethargy, weight loss	Liver damage	Yes	No
Mouse (*Mus musculus*)	A12, C5, D37 and D64	10^5^–1.4 × 10^11^	i.m., i.n., i.pe., i.pu., i.v., i.o., s.c.	No *	Liver, eye and lung damage	Yes	Yes
New Zealand rabbit (*Oryctolagus cuniculus*)	C5	5.7 × 10^5^–1.6 × 10^9^	i.v., i.o.	Eye pathology	Lung and eye damage	Yes	No
Pig (*Sus scrofa*)	C5	1.6 × 10^3^–10^10^	i.v., i.t.	No	Moderate lung damage	Not reported	No
Rat (*rattus norvegicus*)	A12, C5 and D9	5 × 10^7^–3 × 10^11^	i.o. i.pe., s.c.	No	Not reported	Not reported	Yes
Syrian hamster (*Mesocricetus auratus*)	A12, A18, B3, B7, B14, C5 and C6	1.5 × 10^10^–2 × 10^11^	i.i., i.n., i.pu., i.t., s.c., i.v.	Weight loss with some HAdV types ^§^	Liver, lung and airway damage	Yes	Yes
Tree shrew (*Tupaia* *belangeri chinensis*)	B55	5 × 10^5^	i.n.	Weight loss and body temperature increase	Lung damage	Yes	No

^+^ Without mutant HAdVs or engineered oncolytic HAdVs. ^#^ Not all references reported accurate TCID50 values; where appropriate, PFU were converted to TCID_50_ (pfu [mL]/TCID_50_ [mL] = 0.7). * A lethal dose of 3.6 × 10^9^ TCID50 has been reported for cotton rats [76], and ~1.4 × 10^9^ TCID50 for mice [77]. ^§^ Weight loss and lethal challenge have been reported in chemically immunosuppressed Syrian hamsters [55,56]. i.c., intracardial; i.i., intraintestinal; i.m., intramuscular; i.n., intranasal; i.o., intraocular, i.pe., intraperitoneal; i.pu., intrapulmonal; i.t., intratracheal; i.v., intravenous; s.c., subcutaneous.

**Table 2:** Available HAdV animal models—their strengths and limitations.

Model Animal	Strengths	Limitations
Cotton rat	▪ HAdV replicates in the upper respiratory tract and the lungs and causes pneumonia ▪ resembles human EKC ▪ used in many studies → good comparability between studies	▪ difficult animal handling ▪ subtle systemic disease signs, dependent on the infectious dose
Guinea pig	▪ HAdV replicates in the lungs and causes pneumonia ▪ persistent HAdV infection (?)	▪ few studies available → limited comparability between studies
Humanized mouse	▪ persistent HAdV infection ▪ robust clinical readouts like weight loss and lethargy	▪ high expenses ▪ laborious to generate ▪ few studies available → limited comparability between studies
Immunosuppressed hamster	▪ resembles HAdV infection of immunosuppressed humans ▪ weight loss as a robust clinical readout	▪ chemical immunosuppression required
Mouse	▪ countless molecular and genetic tools available ▪ HAdV-induced tumors	▪ mostly non-permissive ▪ subtle clinical disease signs
New Zealand rabbit	▪ resembles human EKC, used in many studies → good comparability between studies ▪ persistent HAdV infection	▪ higher maintenance costs compared to mice and rats ▪ no systemic infection
Pig	▪ HAdV replicates in the lungs and causes pneumonia	▪ animal size and handling ▪ no signs of a clinical disease ▪ few studies available → limited comparability between studies
Rat	▪ HAdV-induced tumors	▪ non-permissive ▪ no signs of a clinical disease
STAT2 KO hamster	▪ increased HAdV replication compared to wild type hamsters ▪ resembles aspects of HAdV infection of immunosuppressed humans	▪ limited availability ▪ animals are immunosuppressed
Syrian hamster	▪ most established HAdV animal model ▪ used in many efficacy studies on HAdV therapeutics → good comparability between studies ▪ various molecular tools available ▪ virus replication in various organs ▪ HAdV-induced tumors	▪ no signs of a clinical disease
Tree shrew	▪ resembles human HAdV-induced pneumonia ▪ robust clinical readouts light weight loss and fever ▪ suitable for studies that assess zoonotic potential of HAdVs (?)	▪ limited availability ▪ few studies available → limited comparability between studies

EKC, (adenoviral) epidemic keratoconjunctivitis; KO, knockout.

## Data Availability

Not applicable.
